# A phase II trial of Cremorphor EL-free paclitaxel (Genexol-PM) and gemcitabine in patients with advanced non-small cell lung cancer

**DOI:** 10.1007/s00280-014-2498-5

**Published:** 2014-06-07

**Authors:** Hee Kyung Ahn, Minkyu Jung, Sun Jin Sym, Dong Bok Shin, Shin Myung Kang, Sun Young Kyung, Jeong-Woong Park, Sung Hwan Jeong, Eun Kyung Cho

**Affiliations:** 1 Division of Hematology and Oncology, Department of Internal Medicine, Gachon University Gil Medical Center, 1198 Guwol-dong, Namdong-gu, Incheon, 405-760 Republic of Korea; 2 Division of Medical Oncology, Department of Internal Medicine, Yonsei University College of Medicine, Seoul, Republic of Korea; 3 Division of Pulmonary and Critical Care Medicine, Department of Internal Medicine, Gachon University Gil Medical Center, Incheon, Republic of Korea

**Keywords:** Non-small cell lung cancer, Gemcitabine, Paclitaxel, Chemotherapy, Genexol-PM

## Abstract

**Purpose:**

Genexol-PM is a Cremorphor EL (CrEL)-free polymeric micelle formulation of paclitaxel that allows higher-dose administration with less hypersensitivity. This study was designed to evaluate the efficacy and safety of Genexol-PM and gemcitabine combination in advanced non-small cell lung cancer patients as a first-line treatment.

**Patients and methods:**

This is a prospective, single-arm, single-center phase II study. Patients with advanced NSCLC received Genexol-PM at 230 mg/m^2^ on day 1 and gemcitabine 1,000 mg/m^2^ on day 1 and day 8 of a 3-week cycle. Six cycles of chemotherapy were planned unless there was disease progression. The primary endpoint was overall response rate.

**Results:**

Forty-three patients received the study drugs with a median of 4 cycles per patient (range 1–6). The overall response rate was 46.5 %. The median progression-free survival was 4.0 months (95 % CI 2.0–6.0 months), and median overall survival was 14.8 months (95 % CI 9.1–20.5 months). The most common toxicities were anemia (*n* = 29, 67 %), asthenia (*n* = 17, 40 %), myalgia (*n* = 16, 37 %), peripheral neuropathy (*n* = 15, 35 %), and diarrhea (*n* = 12, 30 %). The most common grade 3/4 adverse events were neutropenia (*n* = 7, 16 %) and pneumonia (*n* = 5, 12 %). Two patients died of pneumonia and dyspnea.

**Conclusions:**

CrEL-free paclitaxel in combination with gemcitabine demonstrated favorable antitumor activity with little emetogenicities in non-small cell lung cancer patients. However, frequent grade 3/4 toxicities were observed, and safety should be evaluated thoroughly in future studies.

## Introduction

Lung cancer is the most common cause of cancer mortality, and most patients are diagnosed at inoperable advanced stages [1]. In inoperable non-small cell lung cancer, platinum-based doublet chemotherapy is widely accepted as the first-line palliative chemotherapy as it is beneficial to overall survival and quality of life. In the early 2000s, both cisplatin and carboplatin were comparably effective platinum-based regimens when combined with newer chemotherapeutic agents, such as paclitaxel, docetaxel, or gemcitabine [2]. However, platinum’s characteristic side effects including nephrotoxicity and neurotoxicity limit its administration. Based on these considerations, several studies [3–8] reported the results of combination cytotoxic chemotherapy without platinum agents for non-small cell lung cancer. In previous phase III trials [4, 6], paclitaxel 200 mg/m^2^ and gemcitabine 1,000 mg/m^2^ on days 1 and 8 (PG) combination as first palliative chemotherapy had anticancer activity and tolerability similar to carboplatin at an area under the time concentration curve (AUC) of 6 mg and paclitaxel 200 mg/m^2^, and similar activity and better myelotoxicity than carboplatin at AUC of 6 mg and gemcitabine 1,000 mg/m^2^ on days 1 and 8. Compared with these two cisplatin-based regimens, PG combination chemotherapy had similar overall survival and quality of life [8].

Paclitaxel is one of the most commonly used cytotoxic anticancer agents in combination with platinum and possesses anticancer activity as a non-platinum doublet PG regimen. Two major concerns of paclitaxel administration are hypersensitivity reactions and neurotoxicity. Because of paclitaxel’s water insolubility, Cremorphor EL (CrEL) is used as a formulation vehicle, although it has toxic effects such as severe anaphylactoid hypersensitivity reactions, hyperlipidemia, and peripheral neuropathy [9]. To avoid these drawbacks, novel CrEL-free formulations are of interest. Genexol-PM (Samyang Co., Seoul, Korea) is a novel formulation of CrEL-free, polymeric micelle formulation of paclitaxel. In a phase I study [10], Genexol-PM had a maximum tolerated dose of 390 mg/m^2^/3 weeks, higher than that of paclitaxel (175 mg/m^2^/3 weeks). Neutropenia, myalgia, and neuropathy were dose-limiting toxicities. In a phase II trial [11] for non-small cell lung cancer patients, Genexol-PM dose ranging 230–300 mg/m^2^/3 weeks in combination with cisplatin was generally well tolerated and showed significant anticancer activity (overall response rate 37.7 %). The efficacy and tolerability of Genexol-PM as a non-platinum doublet for non-small cell lung cancer patients have not been studied. Given the concerns of platinum side effects and relatively good tolerability of Genexol-PM at higher doses, we designed a phase II study to assess the efficacy and safety of non-platinum doublet of Genexol-PM in combination with gemcitabine in advanced non-small cell lung cancer patients.

## Methods

### Patients

Eligibility criteria included a histologically confirmed diagnosis of advanced non-small cell lung cancer of AJCC 6th stage IIIB and IV. In recurrent cases after curative surgery, a 6-month period after completion of adjuvant chemotherapy was required. No prior chemotherapy or radiotherapy was allowed except for whole brain radiotherapy. Patients were required to be of age ≥18-year old and to have Eastern Cooperative Oncology Group Performance Status of 0–2, at least one measurable lesion, life expectancy more than 3 months, and adequate bone marrow, liver, and renal function. Patients with uncontrolled symptomatic brain metastases were excluded. Written informed consent was obtained from every patient. This study was approved by the Institutional Review Board at Gachon University Gil Medical Center and was registered in clinicaltrials.gov (NCT01770795).

### Study design, treatment and assessment

This was a prospective, single-arm, single-center phase II study. The primary endpoint was an objective response rate assessed by RECIST version 1.0. The secondary endpoints were progression-free survival, overall survival, and safety profiles.

On day 1, Genexol-PM 230 mg/m^2^ diluted in 500 mL of normal saline or 5 % dextrose water was intravenously infused over 3 h. Gemcitabine 1,000 mg/m^2^ was intravenously administered on days 1 and 8. Premedication with antihistamine and steroid was administered as needed. Chemotherapy was repeated with 3-week cycles until completion of intended six cycles, disease progression, consent withdrawal of participant, or occurrence of unacceptable toxicities. When dose reduction was required, study drugs were reduced by 25 %. With grade 3 peripheral neuropathy, Genexol-PM was withheld until the neuropathy recovered to ≤grade 2 and was reduced by 25 % when readministered.

Responses were assessed by contrast-enhanced computed tomography scans of chest and abdomen every 6 weeks during the study drug administration, 3 weeks after the completion or withdrawal of study drugs, and every 8 weeks thereafter. Patients were observed until death or study closure. Toxicities were assessed according to the NCI CTCAE v3.0.

### Statistics

According to Simon’s minimax phase II trial design, to test a null hypothesis with a response rate of 25 % and an alternative of 45 % at α = 5 % and power of 80 %, 41 patients were needed. Forty-five patients were planned to be accrued to allow for a 10 % drop-out rate. Progression-free survival and overall survival were estimated by Kaplan–Meier method. Progression-free survival was calculated from the date of the study drug initiation to the date of documented progression, the last exam, or death. Overall survival was estimated as the time from the date of the first administration of study drug to death or the date of the last follow-up visit.

## Results

### Patients and treatment outcome

Between January 2011 and August 2012, 45 patients were enrolled in the study. Of the 45 patients, two patients withdrew consent before study drug commencement and were excluded from final analysis. Among the final 43 patients who received study treatment, 38 (88 %) were male. The study subjects had a median age of 65 years (range 36–82-year old). Thirteen patients (30 %) were elderly patients over 70-year old. All patients had stage IV disease at the time of study enrollment. Twenty-eight patients (65 %) had adenocarcinoma, eight patients (19 %) had squamous cell carcinoma, and the remaining seven patients had other histologic subtypes. All patients had metastatic disease at the time of study treatment. The result of EGFR mutation analysis was available in 26 patients. Twenty-three patients had wild-type EGFR and three patients had EGFR mutation (two patients with deletion in exon 19 and one with insertion in exon 20). Patient characteristics are described in Table 1.

**Table 1:** Patient baseline characteristics (*n* = 43)

Characteristic	Number (%)
Age (years)	
Median	65
Range	36–82
Sex	
Male	38 (88)
Female	5 (12)
ECOG performance status	
0	22 (51)
1	17 (40)
2	4 (9)
Histology	
Adenocarcinoma	28 (65)
Squamous cell carcinoma	8 (19)
Others	7 (16)
Smoking history	
Smoker	36 (84)
Never smoker	7 (16)
Previous radiotherapy to symptomatic brain metastases	
Yes	4 (9)
No	39 (91)
EGFR mutation	
Wild type	23 (53)
Mutant^*^	3 (7)
Unknown	17 (40)

* Deletion in exon 19 or L858R in exon 21

*ECOG* Eastern Cooperative Oncology Group, *EGFR* epidermal growth factor receptor

The median cycle of treatment administration was 4 (range 1–6). The mean dose intensity was 209 mg/m^2^/3 weeks (90.9 % of preplanned 230 mg/m^2^/3 weeks) for Genexol-PM, and 1853 mg/m^2^/3 weeks (92.7 % of preplanned 2,000 mg/m^2^/3 weeks) for gemcitabine. Sixteen patients (37.2 %) completed the planned six cycles of chemotherapy.

**Fig. 1:**
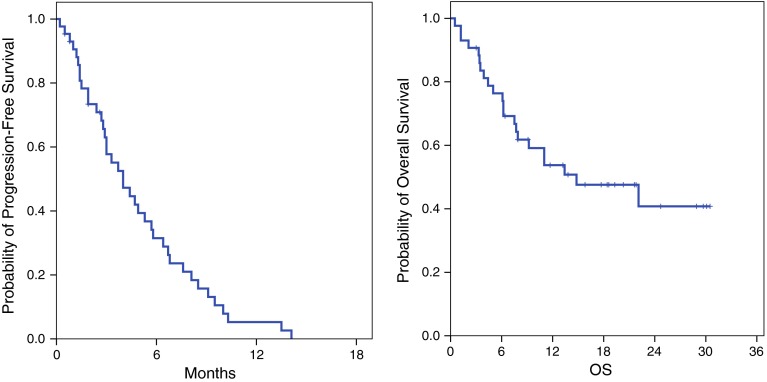
Survival graphs of the 43 patients, **a** progression-free survival, **b** overall survival

Of the 43 patients included in final analysis, treatment response could be assessed in 36 patients. An overall response rate was 46.5 % (95 % CI 31.6–61.4) with 0 CR and 20 PRs. Stable disease (SD) was achieved in eight (18.6 %) patients (Table 2). The reasons for early study discontinuation before the first response evaluation were loss to follow-up (*n* = 1), protocol deviation with palliative radiotherapy (*n* = 1), death (*n* = 2), and prolonged grade 3 adverse events (*n* = 3). After a median follow-up duration of 18.6 months (range 3.0–27.5 months) for living patients, the median PFS was 4.0 months (95 % CI 2.4–5.6 months). The median OS was 14.8 months (95 % CI 3.6–25.9 months) (Fig. 1).

**Table 2:** Response according to RECIST (version 1.0)

Response	Number of patients (95 % CI)
Complete response	0 (0 %)
Partial response	20 (46.5 %, 31.6–61.4)
Stable disease	8 (18.6 %, 7.0–30.2)
Progressive disease	8 (18.6 %, 7.0–30.2)
Withdrawal without evaluation	7 (16.3 %, 5.2–27.3)

### Safety

Among 43 patients, the most common toxicity was anemia (*n* = 29, 85 %) (Table 3). The most common non-hematologic toxic effects were asthenia (*n* = 17), myalgia (*n* = 16), peripheral neuropathy (*n* = 15), diarrhea (*n* = 12), dyspnea (*n* = 12), and anorexia (*n* = 11). Notably, there was no grade 3–4 chemotherapy-induced nausea or vomiting, with only seven patients experiencing grade 1–2 emesis. No hypersensitivity reaction was observed.

**Table 3:** Overview of adverse events

Toxicity	Grade 1/2 No. of patients (%)	Grade 3/4 No. of patients (%)
Hematologic		
Anemia	29 (67)	0 (0)
Neutropenia	0 (0)	7 (16)
Thrombocytopenia	0 (0)	0 (0)
Non-hematologic		
Skin rash	5 (12)	1 (3)
Pruritus	2 (5)	0 (0)
Hypersensitivity reaction	0 (0)	0 (0)
Peripheral neuropathy	13 (30)	2 (5)
Myalgia	14 (33)	2 (5)
Arthralgia	4 (9)	0 (0)
Asthenia	14 (33)	3 (7)
Anorexia	11 (26)	0 (0)
Nausea	4 (9)	0 (0)
Vomiting	3 (7)	0 (0)
Diarrhea	11 (26)	1 (3)
Constipation	5 (12)	0 (0)
Left ventricular dysfunction	0 (0)	1 (3)
Dyspnea	10 (23)	2 (5)
Pulmonary thromboembolism	0 (0)	3 (7)
Pneumonia	0 (0)	5 (12)

The grade 3–4 adverse events (AE) occurred in 22 patients (51.2 %). The most common grade 3–4 hematologic toxicity was neutropenia (*n* = 7) including four patients with neutropenic fever. The most common grade 3–4 non-hematologic toxicities were pneumonia (*n* = 5), followed by asthenia (*n* = 3), pulmonary thromboembolism (*n* = 3), myalgia (*n* = 2), peripheral neuropathy (*n* = 2), diarrhea (*n* = 1), skin rash (*n* = 1), and dyspnea (*n* = 2). In nine patients, the study drug was discontinued because of toxicities. Two patients died during the study period without evidence of disease progression. One patient was found dead upon arrival at an emergency room from sudden dyspnea after 2 cycles of study drugs. The other patient was admitted to an intensive care unit (ICU) to manage grade 4 pneumonia after the first cycle; he was dropped because of prolonged pneumonia treatment and eventually died of cerebral infarction while in the ICU.

## Discussion

In this phase II study, CrEL-free paclitaxel Genexol-PM in combination with gemcitabine demonstrated significant antitumor activity for advanced NSCLC patients, with a partial response rate of 46.5 %. This is the first report of Genexol-PM as a non-platinum combination for NSCLC patients.

The non-platinum doublet of gemcitabine and taxane had similar anticancer activities and different toxicity profiles in lung cancer patients, compared with platinum doublet regimens [3, 6]. Paclitaxel and gemcitabine were compared with combinations of paclitaxel and carboplatin [4], gemcitabine and carboplatin [6], and paclitaxel and vinorelbine [5] in phase III trials. All of the studied regimens possess similar antitumor efficacies, suggesting that the choice among these platinum and non-platinum combination depends on the toxicity profiles. The strengths of the non-platinum regimen lay in less frequent myelotoxicity and emetogenicity than a platinum-based doublet, apparent in the current study, too. This is consistent with the observation of a lower risk of neutropenia than CrEL containing paclitaxel in phase I trials of Genexol-PM [10, 12]. Considering that the elderly lung cancer patients often suffer from increased risk of myelotoxicity and renal toxicity from platinum-based doublet chemotherapy [13], the advantage of non-platinum combination can be further evaluated in this population. Neurotoxicity may be a disadvantage of a non-platinum doublet regimen, as paclitaxel and gemcitabine had a significantly higher risk of peripheral neurotoxicities than gemcitabine and carboplatin [6]. However, in the present study with CrEL-free paclitaxel formulation, higher doses of paclitaxel were well tolerated with similar risks of peripheral neurotoxicities, offering an alternative as a non-platinum doublet.

In line with previous trials of Genexol-PM, our study demonstrated that Genexol-PM enabled administration of higher doses of paclitaxel, as the mean dose intensity of Cremorphor-free paclitaxel was 209 mg/m^2^/3 weeks. Although we cannot make a direct comparison, a study of conventional paclitaxel and gemcitabine for NSCLC patients [6] showed similar adverse event rate of grade 3/4 neutropenia (18 %) and grade 3 peripheral neuropathy (5 %) with a lower dose intensity of paclitaxel (median 170 mg/m^2^/3 weeks). Another trial of paclitaxel and gemcitabine [5] demonstrated a similar dose intensity of paclitaxel and adverse events. In this study, despite the higher paclitaxel dose, the rates of grade 3/4 neutropenia and grade 3 peripheral neuropathy were 18 and 5 %. The overall response rate of this study (46.5 %) seems to be higher than 27.5 and 31 % observed previously [5, 6] with the same regimen of CrEL-based paclitaxel, perhaps resulting from the higher dose intensity of paclitaxel.

The advantage of CrEL-free paclitaxel over conventional paclitaxel was expected to include delivery of higher doses and enhanced tumor distribution [10]. A tendency toward a higher response rate of this study might be related to the higher dose intensity. Expected toxicities such as hematologic toxicity, peripheral neuropathy, and myalgia were favorable with higher doses of paclitaxel administration. CrEL was thought to be related to the hypersensitivity reaction of paclitaxel; therefore, CrEL-free paclitaxel formulation was expected not to produce hypersensitivity reactions. Although no hypersensitivity reactions were observed in a phase I trial, subsequent phase II trials found significant risks of hypersensitivity reactions (seven among 69 patients) or skin rash [11, 14, 15]. Thus, a hypersensitivity reaction may be unavoidable with CrEL-free formulation. We observed no hypersensitivity reactions, although one patient experienced a grade 3 skin rash.

A platinum-based doublet regimen of Genexol-PM for NSCLC patients was studied in a phase II trial [11]. With higher median dose intensity of CrEL-free paclitaxel (252 mg/m^2^/3 weeks) than that of this study, the response rate was comparable (37.7 %) to our study (46.5 %), although the rates of hematologic toxicities and peripheral neuropathy were higher with platinum-based doublet. Moreover, grade 2–4 nausea and vomiting were observed in about 30 % with Genexol-PM and cisplatin, in contrast to 9 % with non-platinum doublet of this study.

Grade 3/4 non-hematologic toxicities were frequent in this study; however, many of the study subjects had poor prognostic factors such as old age, male sex, or heavy smoking history, which might exaggerate the toxicity profiles. All the five events of grade 3–4 pneumonia were bacterial pneumonia, not drug-induced pneumonitis. The possibilities of toxicities from higher doses of paclitaxel itself cannot be completely excluded; therefore, we should be cautiously deliberate to balance the advantages of higher dose of paclitaxel with CrEL-free formulation with the potential harm.

As EGFR tyrosine kinase inhibitor (TKI) was established as first-line treatment during study period, only three subjects with EGFR mutation who could not afford to EGFR TKI were included and EGFR status of the most patients were wild type or unknown. A recent retrospective study [16] reported that EGFR mutant patients had better PFS to taxane-including treatment than a gemcitabine-based regimen. In our study, two patients with sensitive EGFR mutations showed long PFS of 6 and 10 months.

In conclusion, Genexol-PM with gemcitabine showed a comparable efficacy in NSCLC patients with less myelotoxicity and emetogenicity. Given the significant risk of grade 3/4 non-hematologic adverse events, clinicians should be cautious in selection of candidates and further evaluation.

## References

[1] Jung KW, Won YJ, Kong HJ, Oh CM, Shin A, Lee JS (2013). Survival of Korean adult cancer patients by stage at diagnosis, 2006–2010: national cancer registry study. Cancer Res Treat.

[2] Schiller JH, Harrington D, Belani CP, Langer C, Sandler A, Krook J, Zhu J, Johnson DH (2002). Comparison of four chemotherapy regimens for advanced non-small-cell lung cancer. N Engl J Med.

[3] Georgoulias V, Papadakis E, Alexopoulos A, Tsiafaki X, Rapti A, Veslemes M, Palamidas P, Vlachonikolis I (2001). Platinum-based and non-platinum-based chemotherapy in advanced non-small-cell lung cancer: a randomised multicentre trial. Lancet.

[4] Kosmidis P, Mylonakis N, Nicolaides C, Kalophonos C, Samantas E, Boukovinas J, Fountzilas G, Skarlos D, Economopoulos T, Tsavdaridis D, Papakostas P, Bacoyiannis C, Dimopoulos M (2002). Paclitaxel plus carboplatin versus gemcitabine plus paclitaxel in advanced non-small-cell lung cancer: a phase III randomized trial. J Clin Oncol.

[5] Kosmidis PA, Fountzilas G, Eleftheraki AG, Kalofonos HP, Pentheroudakis G, Skarlos D, Dimopoulos MA, Bafaloukos D, Pectasides D, Samantas E, Boukovinas J, Lambaki S, Katirtzoglou N, Bakogiannis C, Syrigos KN (2011). Paclitaxel and gemcitabine versus paclitaxel and vinorelbine in patients with advanced non-small-cell lung cancer. A phase III study of the Hellenic Cooperative Oncology Group (HeCOG). Ann Oncol.

[6] Kosmidis PA, Kalofonos HP, Christodoulou C, Syrigos K, Makatsoris T, Skarlos D, Bakogiannis C, Nicolaides C, Bafaloukos D, Bamias A, Samantas E, Xiros N, Boukovinas I, Fountzilas G, Dimopoulos MA (2008). Paclitaxel and gemcitabine versus carboplatin and gemcitabine in patients with advanced non-small-cell lung cancer. A phase III study of the Hellenic Cooperative Oncology Group. Ann Oncol.

[7] Park SH, Hong J, Kim YS, Kim Y, Kyung SY, An CH, Lee SP, Park JW, Jeong SH, Park J, Cho EK, Shin DB, Lee JH (2008). Phase II trial of weekly docetaxel and gemcitabine for previously untreated, advanced non-small cell lung cancer. Lung Cancer.

[8] Smit EF, van Meerbeeck JP, Lianes P, Debruyne C, Legrand C, Schramel F, Smit H, Gaafar R, Biesma B, Manegold C, Neymark N, Giaccone G (2003). Three-arm randomized study of two cisplatin-based regimens and paclitaxel plus gemcitabine in advanced non-small-cell lung cancer: a phase III trial of the European Organization for Research and Treatment of Cancer Lung Cancer Group–EORTC 08975. J Clin Oncol.

[9] Gelderblom H, Verweij J, Nooter K, Sparreboom A (2001). Cremophor EL: the drawbacks and advantages of vehicle selection for drug formulation. Eur J Cancer.

[10] Kim TY, Kim DW, Chung JY, Shin SG, Kim SC, Heo DS, Kim NK, Bang YJ (2004). Phase I and pharmacokinetic study of Genexol-PM, a cremophor-free, polymeric micelle-formulated paclitaxel, in patients with advanced malignancies. Clin Cancer Res.

[11] Kim DW, Kim SY, Kim HK, Kim SW, Shin SW, Kim JS, Park K, Lee MY, Heo DS (2007). Multicenter phase II trial of Genexol-PM, a novel Cremophor-free, polymeric micelle formulation of paclitaxel, with cisplatin in patients with advanced non-small-cell lung cancer. Ann Oncol.

[12] Lim WT, Tan EH, Toh CK, Hee SW, Leong SS, Ang PC, Wong NS, Chowbay B (2010). Phase I pharmacokinetic study of a weekly liposomal paclitaxel formulation (Genexol-PM) in patients with solid tumors. Ann Oncol.

[13] Kim ST, Park KH, Oh SC, Seo JH, Kim JS, Kim YH, Shin SW (2012). Chemotherapy in patients older than or equal to 75 years with advanced non-small cell lung cancer. Cancer Res Treat.

[14] Lee KS, Chung HC, Im SA, Park YH, Kim CS, Kim SB, Rha SY, Lee MY, Ro J (2008). Multicenter phase II trial of Genexol-PM, a Cremophor-free, polymeric micelle formulation of paclitaxel, in patients with metastatic breast cancer. Breast Cancer Res Treat.

[15] Lee JL, Ahn JH, Park SH, Lim HY, Kwon JH, Ahn S, Song C, Hong JH, Kim CS, Ahn H (2012). Phase II study of a cremophor-free, polymeric micelle formulation of paclitaxel for patients with advanced urothelial cancer previously treated with gemcitabine and platinum. Invest New Drugs.

[16] Park JH, Lee SH, Keam B, Kim TM, Kim DW, Yang SC, Kim YW, Heo DS (2012). EGFR mutations as a predictive marker of cytotoxic chemotherapy. Lung Cancer.

